# Simultaneous Analysis of Irbesartan and Hydrochlorothiazide: An Improved HPLC Method with the Aid of a Chemometric Protocol 

**DOI:** 10.3390/molecules17033461

**Published:** 2012-03-16

**Authors:** Zorica Vujić, Nedžad Mulavdić, Miralem Smajić, Jasmina Brborić, Predrag Stankovic

**Affiliations:** 1 Institute for Pharmaceutical Chemistry, University of Belgrade-Faculty of Pharmacy, Vojvode Stepe 450, 11 000 Belgrade, Serbia; Email: jbrboric@pharmacy.bg.ac.rs; 2 Institute for Pharmaceutical Chemistry, University of Tuzla-Faculty of Pharmacy, Univerzitetska 8, 18 000 Tuzla, Bosnia and Herzegovina; Email: Nedzadmulavdic@yahoo.com (N.M.); smajic.m@hotmail.com (M.S.); 3 Institute of Otorhinolaryngology, Clinical Centre of Serbia, Pasterova 2, 11 000 Belgrade, Serbia; Email: stankovic.pms@sezampro.rs

**Keywords:** **:** HPLC, experimental design, irbesartan, hydrochlorothiazide

## Abstract

Experimental design method was used for HPLC determination of irbesartan and hydrochlorothiazide in combined dosage forms. The traditional approach for optimization of experiments is time-consuming, involves a large number of runs and does not allow establishing the multiple interacting parameters. The main advantages of the experimental design method include the simultaneous screening of a larger number of factors affecting response and the estimation of possible interactions. On the basis of preliminary experiments, three factors-independent variables were selected as inputs (methanol content, pH of the mobile phase and temperature) and as dependent variables, five responses (resolution, symmetry of irbesartan peak, symmetry of hydrochlorothiazide peak, retention factor of irbesartan and retention factor of hydrochlorothiazide) were chosen. A full 2^3^ factorial design, where factors were examined at two different levels (“low” and “high”) was used to determine which factors had an effect on the studied response. Afterwards, experimental design was used to optimize these influent parameters in the previously selected experimental domain. The novelty of our method lies in the optimization step accomplished by Derringer′s desirability function. After optimizing the experimental conditions a separation was conducted on a Supelcosil C_18_ (150 mm × 4.6 mm, 5 μm particle size) column with a mobile phase consisting of methanol-tetrahydrofuran-acetate buffer 47:10:43 v/v/v, pH 6.5 and a column temperature of 25 °C. The developed method was successfully applied to the simultaneous separation of these drug-active compounds in their commercial pharmaceutical dosage forms.

## 1. Introduction

**Figure 1:**
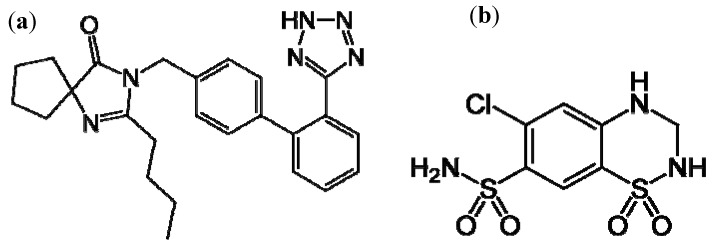
Structural formulae of irbesartan (**a**) and hydrochlorothiazide (**b**).

Irbesartan [IRB, 2-butyl-3-[[2′-(1H-tetrazol-5-yl)[1,1′-biphenyl]-4-yl]- methyl1-3-diazaspiro-[4,4]-non-1-en-4-one, Figure 1(a)] is an angiotensin II blocker. Angiotensin II receptor antagonists represent a relatively new pharmacological class [1] which acts mainly by selective blockade of AT1 receptors and reduces the effects of angiotensin II. They may be used alone or in combination with other antihypertensive or diuretic agents. Hydrochlorothiazide [HCT, 6-chloro-3,4-dihydro-2H-1,2,4-benzothiadiazine-7-sulphonamide-1,1-dioxide, Figure 1(b)] is a diuretic acting on distal convoluted tubule. Because of their synergistic anti-hypertensive action, irbesartan and hydrochlorothiazide are available on the market as a combined dosage form.

A literature survey revealed that there are a number of HPTLC [2,3,4,5], spectrophotometric [6], and spectrofluorometric [7,8], voltammetric [9], capillary zone electrophoretic [10], HPLC [11,12] and LC-MS [13,14] methods for determination of the individual drugs (IRB and HCT) or IRB/HCT in combination with other drugs. Although a number of methods for the determination of irbesartan and hydrochlorothiazide have been reported, there are a limited number of systematic studies for the optimization of separation parameters. 

Some published papers have dealt with the optimization procedure for determination either of IRB (and other angiotensin-II-receptor antagonist) [15,16,17,18] or HCT [19,20], but to the best of our knowledge neither of the already published methods has been related to HPLC method optimization for simultaneous determination of IRB and HCT. The aim of this work was the evaluation of the chromatographic behavior of IRB and HCT using an appropriate experimental design (ED). The main advantage of such approach is simultaneous optimization of influencing factors and response variables which enables prediction of chromatographic retention and postulation of optimum conditions for separation. 

Several statistical approaches could be used for estimating the retention behavior as a function of chromatographic conditions. In this work, full factorial design (FFD) and response surface methodology (RSM) have been applied. Factorial design enables an estimation of investigated factors which have the most importance. Response surface methodology is generally employed in order to provide a description of the response pattern in the region of the studied observations and to assist in finding the region where the optimal response occurs [21].

Marketed tablet formulation (Co-Irda tablets, Nobel Ilac, Turkey) containing 150 mg of irbesartan and 12.5 mg of hydrochlorthiazide have been analyzed. The significant feature of this combination lies in the fact that hydrochlorthiazide is present in minute amounts compared to irbesartan, which makes an analysis more complicated and tedious. In the present paper, a fast, simple and accurate HPLC method has been proposed without the tedious extraction procedure. 

## 2. Results and Discussion

When applying experimental design methodologies, it is advisable to keep the number of variables as low as possible in order to avoid very complex response models and large variability. When the number of influencing factors is up to four, full factorial design (FFD) is recommended. 

The investigation was carried out in several steps. The objective of the first step in the investigation was to perform a screening of the factors that could potentially influence chromatographic retention, thus the independent variables were defined during the preliminary study. Some chromatographic parameters, such as flow rate were excluded as its influence can usually be predicted by common chromatographic theory knowledge. The factors generally selected to optimize the chromatographic separation of ionisable compounds are pH and the content of organic solvent of the mobile phase. The variations of these parameters induce a variation of the degree of ionization and thus affect chromatographic behavior. In addition, column temperature affects retention behavior, thus three factors-independent variables were selected as inputs: methanol content, pH of the mobile phase and temperature. Since a good separation is characterized by good resolution and since run time is very important (from a practical point of view), five responses were chosen as dependent variables: resolution (k_R_), symmetry of the irbesartan peak (Sym_IRB_), symmetry of the hydrochlorothiazide peak (Sym_HCT_), retention factor of irbesartan (Rt_IRB_) and retention factor of hydrochlorothiazide (Rt_HCT_).

**Figure 2:**
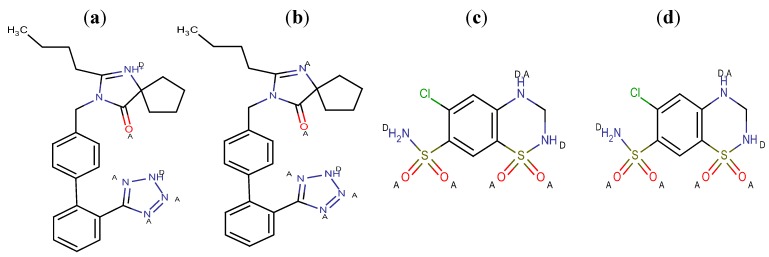
Proton acceptor (A) and donor groups (D) of IRB and HCT at pH 4 (**a**,**c**) and pH 6 (**b**,**d**).

**Figure 3:**
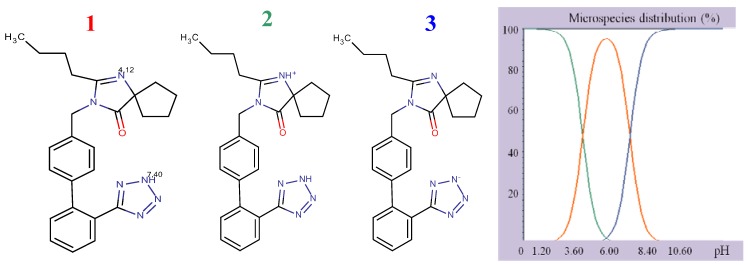
Structural formulae of ionized IRB.

**Figure 4:**
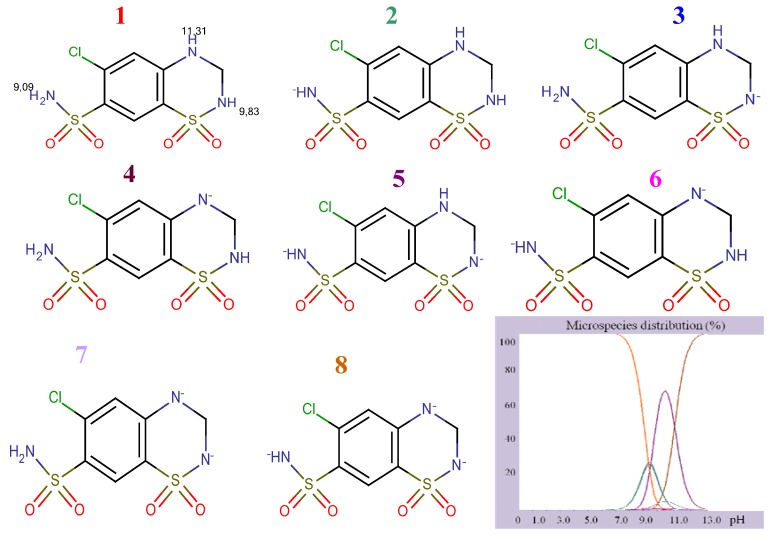
Structural formulae of ionized HCT.

The presence of several functional groups in the molecular structures, such as biphenyl, imidazole and benzene (Figure 1), makes a RP-HPLC method with PDA detection suitable for the determination. Since the RP-HPLC method is based on using a polar mobile phase, a complete description of the ionization profile of the examined substances has been used for the evaluation of retention behavior and also for the separation. The degree of ionization of the drug strongly affects solubility and retention. Additionally, the knowledge of dissociation constant of ionisable compounds at different pH values and the solvent composition is also significant to determine the optimal separation conditions in reversed phase liquid chromatography (RP-LC). Considering the chemical structures, it is possible to establish a number of proton acceptor and donor groups (Figure 2), to assume ionized structures of IRB and HCT (Figure 3 and Figure 4) and degrees of ionization depending on the pH (Table 1 and Table 2). 

**Table 1:** Percentage of ionization of IRB depending on pH.

pH	1	2	3	4	5	6	7	8	9	10	11
%-1	0.08	0.76	7.11	43.34	88.12	94.96	71.43	20.05	2.45	0.25	0.03
%-2	99.92	99.24	92.89	56.64	11.52	1.24	0.09	0	0	0	0
%-3	0	0	0	0.02	0.35	3.79	28.47	79.94	97.55	99.75	99.97

**Table 2:** Percentage of ionization of HCT depending on pH.

pH	1	2	3	4	5	6	7	8	9	10	11	12	13
%-1	100	100	100	100	99.99	99.92	99.19	92.33	51.53	4.55	0.05	0	0
%-2	0	0	0	0	0	0.04	0.41	3.78	21.11	18.65	2.16	0.06	0
%-3	0	0	0	0	0	0.04	0.39	3.62	20.21	17.85	2.07	0.05	0
%-4	0	0	0	0	0	0	0.02	0.15	0.84	0.74	0.09	0	0
%-5	0	0	0	0	0	0	0	0.1	5.78	51.04	59.03	15.56	1.84
%-6	0	0	0	0	0	0	0	0	0.08	0.74	0.86	0.23	0.03
%-7	0	0	0	0	0	0	0	0.01	0.42	3.70	4.28	1.13	0.13
%-8	0	0	0	0	0	0	0	0	0.03	2.72	31.47	82.97	98

As it can be seen, IRB has two pKa values (4.12, imidazole nitrogen; 7.40, tetrazole nitrogen) and at pH 4 it is present as a mixture of molecular (43.34%) and monoprotonated (56.64%) forms. The structure of HCT has three pKa values (9.09, sulphonamide group; 9.83 cyclic sulphonamide; 11.31, secondary amine). At higher pH values, HCT is present as a mixture of different deprotonated species. Having in mind the acid-base properties of IRB and HCT, the pH interval from 4.0 to 6.5 was chosen for further investigation. In this pH interval, IRB is partially ionized and HCT is completely unionized and under these conditions the following order of retention could be expected: HCT, then IRB. The retention of a substance is a function of the volume fraction of the organic modifier in the mobile phase. Taking into account the variation of the retention factors of compounds with polarity of the mobile phase, a range of methanol concentrations from 35% to 55% was selected for investigation.

The analysis of IRB and HCT was started on a non-polar stationary phase (Supelcosil C_18_ column, 150 mm × 4.6 mm, 5 μm particle size) with the mobile phase consisting of methanol-tetrahydrofuran- acetate buffer (pH of mobile phase was adjusted to 4 with acetic acid). The column temperature was set at 35 °C and the flow rate at 0.75 mL/min. Acceptable separation was achieved with methanol- tetrahydrofuran-acetate buffer mixtures ranging from 35:10:55 to 55:10:35 (v/v/v), but peak shape and run time needed to be improved. In order to evaluate the effect of the most important factor, a 2^3^ full factorial design (FFD) with three replicates at the zero level was chosen. The experimental data was coded in order to follow the significance of factors in an easier way. Factors and their “low” (−1), “high” (+1) and “zero” (0) values are presented in Table 3. 

**Table 3:** Factors and levels.

Factors		Factor levels		
		(−)	(+)	(0)
A	CH_3_OH	35	55	45
B	pH of mobile phase	4.0	6.5	6.0
C	T (°C)	25	50	35

A two-level factorial design runs and three replicates of the central point needed 11 runs to complete a whole factorial design. As dependent variables, five responses were chosen: Resolution (k_R_), symmetry of irbesartan peak (Sym_IRB_), symmetry of hydrochlorothiazide peak (Sym_HCT_), retention factor of irbesartan (Rt_IRB_) and retention factor of hydrochlorothiazide (Rt_HCT_). The matrix of experiments and results obtained as an average value of three runs are presented in Table 4.

**Table 4:** Factorial design matrix and results of experiments.

Factors			Results				
A	B	C	k_R_	Sym_IRB_	Sym_HCT_	Rt_IRB_	Rt_HCT_
1	−1	−1	7.11	1.08	1.24	3.727	2.355
−1	−1	1	25.2	0.97	1.21	9.762	2.48
0	0	0	5.14	1.26	1.26	3.312	2.412
−1	−1	−1	27.56	0.97	1.53	14.567	2.79
1	−1	1	5.8	1.13	1.29	3.225	2.258
0	0	0	5.95	1.28	1.28	3.68	2.53
0	0	0	5.33	1.28	1.29	3.447	2.49
1	1	1	2.52	1.48	1.3	2.605	2.263
1	1	−1	2.65	1.21	1.31	2.847	2.37
−1	1	1	11.56	1.49	1.2	5.352	2.51
−1	1	−1	14.68	1.52	1.46	8.145	2.867

Appropriate calculations were done with the Design-Expert 7.0 software (Stat-Ease Inc. Minneapolis, MN, USA). A second-order interaction model was suggested as a model of relationship between input and output and is presented by Equation (1):

y = b_0_ + b_1_A + b_2_B + b_3_C + b_12_AB + b_13_AC + b_23_BC + b_123_ABC (1)

where *b_0_* is the intercept, *b_i_*(*b_1_*, *b_2_*, *b_3_*), *b_ij_* (*b_12_*, *b_13_*, *b_23_*) and *b_ijk_* represent the regression coefficient and *A*,*B*,*C* represent independent variables. The calculated coefficient and model of polynomial regression is presented in Table 5.

**Table 5:** Model of coefficients.

	b_0_	b_1_	b_2_	b_3_	b_12_	b_13_	b_23_	b_123_
k_R_	12.14	−7.62	−4.28	−0.87	2.35	0.51	0.052	0.24
Sym_IRB_	1.23	−0.006	0.19	0.036	−0.074	0.044	0.024	0.031
Sym_HCT_	1.32	−0.032	0	−0.068	0.02	0.078	0	−0.015
Rt_IRB_	6.28	−3.18	−1.54	−1.04	1.17	0.86	0.28	−0.22
Rt_HCT_	2.49	−0.18	0.016	−0.11	−0.011	0.058	−0.007	0.0046

The repetition of the central experimental point provided a precise estimation of the experimental errors and the measure of the adequacy of the models (lack of fit). The results were analysed by ANOVA method and the results are presented in Table 6.

The lack of fit test was determined by performing Fischer-F test. The high value of F with a very low probability (only model terms with corresponding *p*-value lesser than 0.05 are significant at 95% confindence level) implies that there was no evidence of the models lack-of-fit and the models could be accepted as an adequate representation of the data. In addition, the values of R^2^ and R^2^ adjusted taking into account the degrees of freedom indicated that the regression model fits the data well. The exception is a model which considers the retention time of HCT. Since *p* is greater than 0.05, the model needs reduction in order to improve the relationship between parameters.

**Table 6:** Statistical parameters of models obtained by ANOVA.

	*SS_models_*	*SS_models/df_*	*F*	*p*	*R^2^*	*R^2^_adj_*
k_R_	663.23	94.75	537.93	0.0019	0.9995	0.9976
Sym_IRB_	0.38	0.055	409.59	0.0024	0.9993	0.9969
Sym_HCT_	0.098	0.014	59.97	0.0165	0.9953	0.9787
Rt_IRB_	126.28	18.04	520.54	0.0019	0.9995	0.9975
Rt_HCT_	0.37	0.053	14.70	0.0652	Not significant	

The data collected from the performed FFD design led to the following conclusions: it was noticed that methanol content in the mobile phase and pH have the largest influence on k_R_, Rt_IRB_ and less influence on the other responses. This influence had a minus sign, which means that the higher pH values and percentage of methanol in the mobile phase will reduce resolution and retention time of IRB. At the same time, the temperature of the column has a positive effect on Sym_IRB_ and a negative effect on Sym_HCT_. Since the selected responses were not affected in the same manner an additional optimization procedure was needed.

In order to get the best chromatographic performance, the multicriteria methodology was employed by means of Derringer′s desirability function [22]. It is based on constructing desirable ranges for each response (individual desirable function, d_i_) and establishing an overall desirability function (the Derringer desirability function). The Derringer′s desirability function is defined as the geometric mean of individual desirability functions and can be expressed by Equation (2):

D = (d^p1^1 × d^p2^2 × .... d^pn^n)^1/n^ (2)

where n is the number of responses, and p^n^ is the weight of the responses. Weight of the response is the relative importance of each individual functions d_i_ and may range from 0.1 to 10. With a weight of 1, d_i_ varies in a linear way. In this study, weights equal to 1 was selected.

Individual desirability functions range from 0 (undesired response) to 1 (a fully desired response). A value of D close to 1 means that the combination of different criteria is globally optimal. If any of the responses or factors falls outside their desirability range, the overall function becomes zero. There are several ways for calculating the desirability function depending on the goal desired. The meanings of the goal parameters are:

**Maximum**:

0 if response < low value; 0 ≤ d_i_ ≤ 1 as response varies from low to high; d_i_ = 1 if response > high value

**Minimum**:

1 if response < low value; 1 ≥ d_i_ ≥ 0 as response varies from low to high; d_i_ = 0 if response > high value 

**Target**:

0 if response < low value; 0 ≤ d_i_ ≤ 1 as response varies from low to target; 1 ≥ d_i_≥ 0 as response varies from target to high; d_i_ = 0 if response > high value

**Range**:

0 if response < low value; d_i_ = 1 as response varies from low to high; d_i_ = 0 if response > high value.

The goals of multicriteria optimization for each response in this paper are shown in Table 7.

**Table 7:** Criteria for multivariate optimization of the individual responses.

	Goal	Lower limit	Upper limit	Weight	Importance
Percentage of methanol	In range	−1	1	1	3
pH	In range	−1	1	1	3
T	In range	−1	1	1	3
k_R_	Is target = 3	2.52	27.56	1	3
Sym IRB	In range	0.97	1.52	1	1
Sym HCT	In range	1.2	1.53	1	1
Rt IRB	Is target = 5	2.605	14.567	1	5
Rt HCT	In range	2.258	2.867	1	5

**Figure 5:**
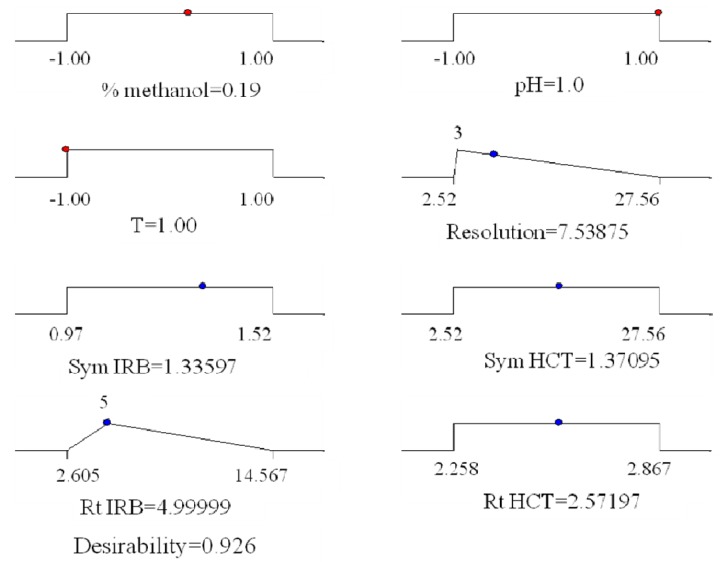
Graphical representation of the constraints accepted fot the determination of global desirabilty and obtained optimal conditions.

Desirability function calculations were performed using Design-Expert^®^ 7.0. Obtained results are graphically presented (Figure 5).

**Figure 6:**
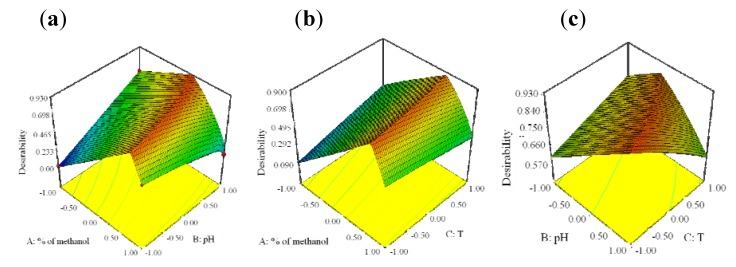
3-D plots of the Derringer’s desirability function in correlation with a variation of methanol content and pH (**a**), methanol content and temperature (**b**) and pH of mobile phase and temperature of column (**c**).

For better visualization of the results, the global desirability function D was presented in a form of a three-dimensional plot and presented in Figure 6.

**Figure 7:**
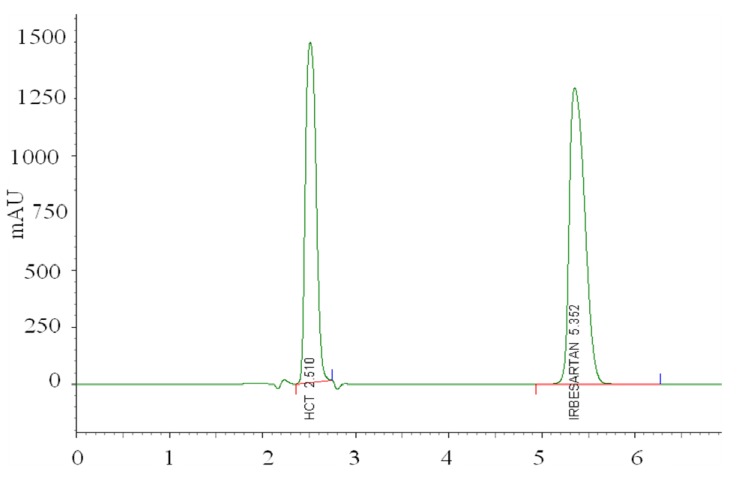
LC-PDA chromatogram of hydrochlorothiazide and irbesartan taken under optimized experimental conditions.

The coordinates related to the functions maximum are selected as the best operating conditions. The best chromatographic conditions are achieved with coded values of: % of methanol 0.19, pH of mobile phase 1 and column’s temperature −1, *i.e.*, with mobile phase methanol:tetrahydrofuran:acetate buffer from 47:10:43 v/v/v, pH 6.5 and column′s temperature 25 °C. The representative chromatogram taken under these conditions is represented in Figure 7. 

After setting the optimal conditions, the proposed method was validated. Interfering peaks were not detected at the retention time of IRB and HCT, indicating the good selectivity of the method. Linear dependence of the peak areas *versus* concentration was determined for the proposed ranges. Parameters of the linear regression equations were calculated and are presented in Table 8. The statistical significance of the intercept was tested using Student’s t-test. The limit of detection (LOD) and limit of quantification (LOQ) were calculated as LOD = 3σ/S and LOQ = 10σ/S, where σ is the standard deviation of the response and S the intercept determined from the corresponding calibration curve.

**Table 8:** Statistical parameters for individual calibration curves.

Parameter	IRB	HCT
Concentration range (mg/mL)	0.08–0.4	0.02–0.1
Slope	2.448	8.609
Intercept	0.035	0.0732
R^2^	0.9976	0.9954
Sa	0.007	0.076
Sb	0.812	0.021
t *	1.872	1.256
LOD	0.02	0.006
LOQ	0.06	0.018

* t_tab_ = 2.447 (*p* = 0.05, f = 6).

Precision of the procedure was assessed by analyzing nine solutions containing known quantities of the investigated compounds. Law values of relative standard deviation for repeatability, RSD < 2.5%, and high recovery (Table 9) indicate very good precision of the proposed method.

**Table 9:** Precision of the RP-HPLC method.

Sample	Injected (mg/mL)	Found (mg/mL)	RSD (%)
Irbesartan	0.120	121.29 ± 0.13	1.26
	0.160	160.09 ± 0.15	1.35
	0.190	191.75 ± 0.21	1.54
Hydrochlorothiazide	0.040	0.040 ± 0.91	0.44
	0.050	0.050 ± 0.82	0.53
	0.060	0.060 ± 0.87	0.59

The applicability of the proposed method was examined by analysing commercially available Co-Irda tablets.

## 3. Experimental

### 3.1. Drugs and Reagents

The irbesartan and hydrochlorothiazide standards and Co-Irda tablets (Nobel Ilac, Istanbul, Turkey) consisting of 150 mg IRB and HCT were obtained from Zada Pharmaceuticals d.o.o. (Tuzla, Bosnia and Herzegovina). 

All solvents: methanol, tetrahydrofuran and sodium-acetate (purchased by Chromosol, Sigma-Aldrich, Munich, Germany), and acetic acid (Fluka, Eindhoven, The Netherlands) were of a grade suitable for high-performance liquid chromatography analysis. The HPLC analyses were done by using a Thermo Finnigan Surveyor chromatographic system equipped with a PDA detector and sample injections were made through an injector valve with a 5 μL sample loop. Separations were performed on a Supelcosil C_18_ column (150 mm × 4.6 mm, 5 μm particle size) with detection at 271 nm. Mobile phases were prepared according to the plan of the experiments given in Table 4. The resulting mobile phases were degassed and vacuum filtered through a 0.45 μm membranes filter (Alltech Associates, Lokeren, Belgium). The flow rate was 0.75 mL/min.

### 3.2. Software

Experimental design, statistical analysis and desirabiity function calculation were performed by using MarvinSketch 5.8.2 (Chem Axon Ltd., Somerville, MA, USA, and Budapest, Hungary) and Design-Expert^®^ 7.0 (Stat-Ease Inc.).

### 3.3. Solutions

Stock solutions were prepared by dissolving standard substances in methanol to obtain concentrations of 0.8 mg/mL for IRB and 0.2 mg/mL for IRB. 

#### 3.3.1. Solutions for Method Optimization

Stock solutions were diluted with methanol to obtain a concentration of 0.08 mg/mL of IRB and 0.02 mg/mL of HCT.

#### 3.3.2. Standard Solutions for Linearity Testing

For the calibration curves, a series of eight solutions were prepared from stock solution in the concentration range from 0.08 to 0.4 mg/mL for IRB and from 0.02 to 0.1 mg/mL for HCT.

#### 3.3.3. Solutions for Accuracy Testing

The laboratory mixture containing placebo, IRB and HCT was prepared in the ratio related to the investigated tablets. For the quantiative analysis, three solutions corresponding to 80%, 100% and 120% to those in tablets were prepared.

#### 3.3.4. Solutions for Estimating Precision

In order to estimate precision, three series (0.16, 0.24 and 0.32 mg/mL for IRB; 0.04, 0.06 and 0.08 mg/mL for HCT) were prepare with ten solutions for each of the concentrations.

#### 3.3.5. Sample Solutions

A tablet mass which corresponds to 150 mg IRB and 12.5 mg HCT was dissolved in 100 mL volumetric flask with methanol, placed into a ultrasonic bath and filtrated. 2 mL of filtrate were diluted with methanol to 10 mL. 

## 4. Conclusions

Experimental design methodology was used for simultaneous HPLC determination of irbesartan and hydrochlorothiazide in combined dosage forms. The significant feature of these combinations lies in the fact that hydrochlorothiazide is present in minute amounts compared to irbesartan which makes for a more complicated and tedious analysis. 

The chemometric approach for optimization of chromatographic separation of irbesartan and hydrochlorothiazide has been demonstrated. The chemometric methodology chosen for the particular objectives was very successful in the retention behavior exploration. Since there was a mix of linear responses with different targets, Derringer′s desirability function was applied. After defining a global desirability according to the accepted constraints, optimal chromatographic conditions were established. 

The proposed HPLC method was validated according to ICH guidelines. From the study of validation parameters, it was observed that the method is specific, accurate, precise, reproducible and is not time-consuming (run time is less than six minutes). Since there was no interference from other components present in the dosage forms, complicated procedures for extraction were not required. The results obtained in this study corroborate that the proposed HPLC method can be used for routine quantitative analyses of the investigated compounds in a mixture or for their individual determination in pharmaceutical dosage forms.
